# Neurodynamic techniques vs. desensitization maneuvers in the manual therapy management of moderate carpal tunnel syndrome among women: randomized clinical trial with 1-year follow-up

**DOI:** 10.3389/fpain.2026.1792933

**Published:** 2026-07-23

**Authors:** Mohamed I. Mabrouk, Sahar A. Abdalbary, Ehab A. A. El-Shaarawy, Sami Elmahgoub, Aseel Aburub, Mohammad Z. Darabseh, Alexandra Makai, Pongrác Ács

**Affiliations:** 1 Department of Physiotherapy, Faculty of Allied Medical Sciences, Applied Science Private University, Amman, Jordan; 2 Department of Orthopaedic Physical Therapy, Faculty of Physical Therapy, Nahda University, Beni Suef, Egypt; 3 Department of Anatomy and Embryology, Faculty of Medicine, Cairo University, Giza, Egypt; 4 Department of Physiotherapy, School of Rehabilitation Sciences, The University of Jordan, Amman, Jordan; 5 Institute of Physiotherapy and Sports Science, Faculty of Health Sciences, Pecsi Tudomanyegyetem Egeszsegtudomanyi Kar, Pécs, Hungary

**Keywords:** carpal tunnel syndrome, cervical spine, desensitisation, manual therapy, nerve conduction 3

## Abstract

**Objective:**

This study aimed to compare the clinical and electrophysiological effects of neurodynamic techniques vs. desensitization maneuvers in women with moderate carpal tunnel syndrome (CTS).

**Design:**

Randomized clinical trial. The study included 380 female participants with moderate carpal tunnel syndrome, aged between 45 and 55 years, who were randomly assigned to a neurodynamic technique group or a desensitization maneuvers group. Neurodynamic techniques in one group and desensitization in the other group were conducted for 3 months.

**Outcome:**

Measures were nerve conduction, pain, symptom severity, and functional status using the Boston Carpal Tunnel Questionnaire, and cylindrical and pincer grip strength before treatment and after 3 months of treatment. One-year follow-up for pain, symptom severity, and functional status using the Boston Carpal Tunnel Questionnaire.

**Results:**

Both groups demonstrated significant within-group improvements in nerve conduction, pain, symptom severity, and functional status (*P* < 0.001). Between-group analysis showed greater improvements in sensory and motor nerve conduction, pain, symptom severity, and functional status in the desensitization group (*P* < 0.001). Improvements in cylindrical and pincer grip strength were significant only in the desensitization group. Clinical gains in pain and function were maintained at 1-year follow-up.

**Conclusion:**

Both interventions were effective in women with moderate CTS; however, desensitization maneuvers demonstrated superior improvements in neurophysiological outcomes, pain, and function over 12 weeks, with sustained benefits at 1-year follow-up.

**Clinical Trial Registration:** http://www.clinicaltrials.gov/, NCT05150912.

## Introduction

Carpal tunnel syndrome is a symptom complex consisting of numbness in the median nerve distribution, typically involving the thumb, index finger, and middle finger. Symptoms frequently occur when performing tasks that require wrist extension while driving for example, and at night (1). Carpal tunnel syndrome has the potential to substantially limit the performance of activities of daily living in some individuals (2).

Carpal tunnel syndrome affects 1%–5% of the general population and is a leading cause of work disability. In the syndrome of carpal tunnel, the ages of 45–54 are the peak incidence years, and women are three times more likely than men to have Carpal tunnel syndrome (3). Most carpal tunnel cases are idiopathic, and extrinsic risk factors include trauma, radiocarpal arthritis, and repetitive work-related activities (4).

The treatment for carpal tunnel syndrome may be conservative or surgical. Conservative treatment is the first-choice approach in most cases, even with surgical recommendations or when surgery is contraindicated (5). Conservative treatment can include splinting, corticosteroid injections, and manual therapy, which includes exercises and mobilization, and most treatment of carpal tunnel syndrome is localized treatment for the hand (5).

The authors' knowledge, there is no standard conservative treatment for carpal tunnel syndrome, it seems reasonable to use neurodynamic techniques in treatment because they can restore the dynamic balance between the relative motion of the nerve and surrounding tissues, improving the sliding of the median nerve, which may improve the neurophysiological function of the median nerve and reduce symptoms of patients (6).

Many previous studies focused on the treatment of carpal tunnel syndrome through localized treatment for the hand, such as ultrasound, splinting, and exercises. Although recent evidence suggests that carpal tunnel syndrome represents a complex pain syndrome that includes sensitization of the central nervous system, it could play a key role in the development of sensory symptoms in the carpal tunnel and can determine therapeutic strategies for this condition (7).

Desensitization maneuvers results are significant improvements for pain and function at 1 and 3 months (8). By incorporating the physiology of pain and sensitization techniques into its therapeutic approach, manual physical therapy can alter mechanisms of sensitization (7). Consequently, 61% of people with carpal tunnel syndrome attempted to postpone surgery (9). Many trials with short-term and long-term results are needed to further determine the most effective technique for the treatment of carpal tunnel syndrome.

Although neurodynamic techniques and desensitization-based manual therapy have each demonstrated benefits in CTS management, existing trials have evaluated these approaches independently, often with short-term outcomes and limited neurophysiological assessment. Moreover, emerging evidence suggests that CTS involves both peripheral nerve pathology and central sensitization mechanisms, which may influence symptom persistence and treatment response. To date, no randomized clinical trial has directly compared neurodynamic techniques with desensitization maneuvers while incorporating electrophysiological measures and long-term follow-up. Therefore, this study aimed to determine whether a treatment strategy targeting central and peripheral sensitization provides superior clinical and neurophysiological outcomes compared with a predominantly peripheral nerve–focused approach.

## Methods

### Ethical considerations

All study procedures were performed in accordance with the Helsinki Declaration of Human Rights of 2013 (Registered at http://www.clinicaltrials.gov/ under NCT05150912). Participants gave written informed consent before data collection began. The first patient was enrolled in this trial in January 2022.

### Study design

Randomized clinical trial with 1-year follow-up. A total of 380 women (45–55 years) with electrophysiologically confirmed moderate CTS were randomly assigned to a neurodynamic technique group or a desensitization maneuver group. Interventions were delivered for 12 weeks.

To be suitable for this study, they were required to meet the following inclusion criteria: pain and paresthesia in the median nerve distribution unilaterally; positive Tinel sign; and positive Phalen sign. Symptoms needed to persist for 1 year prior. Electrodiagnostic examination revealed sensory and motor deficits at median nerve conduction (median nerve sensory conduction velocity less than 4.0 m/s and median nerve distal motor latency greater than 4.20 ms), according to the guidelines of the American Association of Electrodiagnostic Medicine American Academy of Physical Medicine and Rehabilitation (10). Severity of carpal tunnel syndrome was classified as follows: minimal (abnormal segmental, comparative tests only); moderate (abnormal median nerve sensory velocity conduction and distal motor latency); or severe (absence of median nerve sensory velocity conduction and abnormal distal motor latency) (10). All participants had moderate severity of carpal tunnel syndrome.

Participants were excluded if they were male; had any sensory or motor deficits in either the ulnar or radial nerve; previous injury or steroid injections; coexisting cervical radiculopathy; cervical, shoulder, elbow, or hand trauma; systemic disease-causing carpal tunnel syndrome (e.g., diabetes mellitus, thyroid disease); musculoskeletal conditions (e.g., rheumatoid arthritis or fibromyalgia); pregnancy (11). All subjects signed an informed consent form before inclusion in the study.

The sample size was estimated based on preliminary results for 30 participants. To determine the sample size, the study used the following variables: pain, symptom severity, and functional status of the Boston Carpal Tunnel Questionnaire. An alpha of 0.05 and statistical power of 0.80 were utilized. Based on this calculation, the aim was to recruit approximately 190 patients in each group.

### Protocol

#### Randomization and allocation

This trial was conducted in accordance with CONSORT guidelines for randomized clinical trials. Eligible patients were randomly allocated to two parallel groups: neurodynamic techniques (1) or desensitization maneuvers (2). Concealed allocation was conducted using a computer-generated randomized table of numbers created by a nurse not involved in the trial, and who did not participate in the analysis or interpretation of the results. Individual and sequentially numbered index cards with random assignments were prepared. The index cards were folded and placed in sealed opaque envelopes. Another nurse opened the envelope and proceeded with treatment according to the group assignment.

The outcome assessor, neurophysiologist, and data analyst were blinded to group allocation; due to the nature of the interventions, treating therapists and participants could not be blinded. The diagnosis was made by an orthopedist before the patient was assigned to the research group. The nerve conduction study was performed by a neurophysiologist with more than 15 years of experience in the field and who was blinded to the patient group. Physiotherapists who performed all treatment procedures for both groups had 26 years of experience with CTS and did not know anything about the patient group.

The physiotherapist who performed the assessment for pain, Boston Carpal Tunnel Questionnaire Symptoms severity scale, Boston Carpal Tunnel Questionnaire Functional status scale, Cylindrical Grip, and pincer grip before and after treatment, and at 1-year follow-up for both groups had 11 years of experience with carpal tunnel syndrome patients and was blinded to patient group. All participants, before and after 12 weeks of therapy, and at 1 year of follow-up, were re-examined by the same orthopedist and neurophysiologist.

### Interventions

Patients allocated to the neurodynamic techniques group received 36 sessions, three sessions a week for 12 weeks, as described by Wolny and Linek (12). The patients underwent neurodynamic techniques on the median nerve in a lying position, arm adduction to 90°, arm external rotation, wrist and finger extension, forearm supination, and elbow extension. In this sequence, gliding and tension were performed in the proximal and distal directions:

- One-direction proximal glide mobilization (movement: elbow extension, large amplitude of motion)
- One-direction distal glide mobilization (movement: wrist extension, large amplitude of motion)
- One-directional proximal tension mobilization (movement: elbow extension, small amplitude of motion at the end of movement)
- One-directional distal tension mobilization (movement: wrist extension, small amplitude of motion)

Patients allocated to the desensitization maneuver group received 12 sessions, once per week, including maneuvers targeted to the cervical spine and to the areas anatomically related to potential entrapment of the median nerve (i.e., shoulder, elbow, forearm, wrist, and fingers) for 30 min, as described by Fernandez-De-Las-Penas et al. (11). The desensitization maneuvers consisted of several soft tissue mobilization and nerve/tendon gliding exercises, including manual techniques directed at the anatomical sites of entrapment of the median nerve. The intervention was applied to the cervical region in the form of lateral glides, and posteroanterior pressure was directed to the mid-cervical spine. In addition, soft tissue interventions targeting the scalene muscles, costoclavicular space, pectoralis minor, biceps brachii muscle, bicipital aponeurosis, pronator teres, transverse carpal ligament, palmar aponeurosis, and lumbrical muscles were performed. In the final step, the physical therapist performed cervical spine stretching exercises by setting the position of the upper fibers of the trapezius muscle, the upper fibers of the scalene muscles, and stretching the elevator scapulae muscle. Although the total number of sessions differed between groups, treatment frequency and duration were based on previously published protocols for each intervention. The neurodynamic protocol required higher session frequency to achieve graded nerve mobilization, whereas desensitization maneuvers emphasize central and peripheral modulation with longer-lasting effects per session. This design reflects the real-world clinical application of each technique rather than dose-matched experimental conditions.

### Outcome measures

The primary outcome was the change in pain intensity measured by the Numerical Pain Rating Scale (NPRS). Secondary outcomes included median nerve sensory and motor conduction parameters, symptom severity, and functional status assessed using the Boston Carpal Tunnel Questionnaire (BCTQ), and cylindrical and pincer grip strength. Outcomes measured were recorded 2 days before treatment; after the last session, the participants were assessed for nerve conduction, the numerical pain rating scale, the Boston Carpal Tunnel Questionnaire, and the strength of the cylindrical and pincer grips. At 1-year follow-up, participants were assessed using the numerical pain rating scale, Boston Carpal Tunnel Questionnaire, and strength of cylindrical and pincer grips. The median nerve conduction study was performed in an electroneurography laboratory by an experienced neurophysiologist with more than 15 years of experience.

Pain was assessed using a numerical pain rating scale (0 = no pain, 10 = maximum pain) (13). The participants were asked to rate the worst pain in the 2 days before the treatment, after 3 months of treatment, and at 1 year of follow-up after the treatment.

To assess symptom severity and physical capacity, the Boston Carpal Tunnel Questionnaire (14) was used before the treatment, for 2 days before and immediately after 3 months of treatment, and at 1 year of follow-up. The strength of the cylindrical and pincer grips was measured using Jamar Dynamometers (15), measured in kilograms. The strength of the cylindrical and pincer grips was measured before the treatment for 2 days, immediately after 3 months of treatment, and at 1 year of follow-up.

### Sample size

Sample size was based on a previous power calculation using standard deviations obtained from preliminary data analysis, which determined that 380 patients in two groups would provide 80% power to detect a difference of 1.1 mm between patients on the 10 mm Numerical Pain Rating Scale (*α* = 0.05).

### Statistical analysis

Statistical analysis was performed using SPSS software (version 18.0, Chicago, IL). Baseline demographic variables were compared between both groups using an independent *t*-test for age and body mass index, and a Chi-square test for side of the dominant hand and the side of the symptomatic hand. Because the participants received intervention only for one hand, and it was the affected hand, used mean and standard deviation, with time (baseline, 3 months after therapy, and 1 year after therapy) was used to compare both groups in repeated measurements for nerve conduction study pain, Boston Carpal Tunnel Questionnaire, and strength of the cylindrical and pincer grips. Tukey's *post-hoc* test was used in the analysis of between-group differences. Significant results were presented as mean differences and 95% confidence intervals. For all analyses, the *P*-value was considered significant at <0.05. An intention-to-treat analysis was performed. Missing data at the 1-year follow-up were handled using last observation carried forward. Repeated-measures ANOVA was applied to evaluate time × group interactions. Effect sizes (Cohen's *d*) and 95% confidence intervals were calculated for between-group differences. Between-group effect sizes were calculated using Cohen's *d* based on pooled standard deviations. Effect sizes were interpreted as small (0.2), moderate (0.5), and large (≥0.8).

## Results

**Figure 1:**
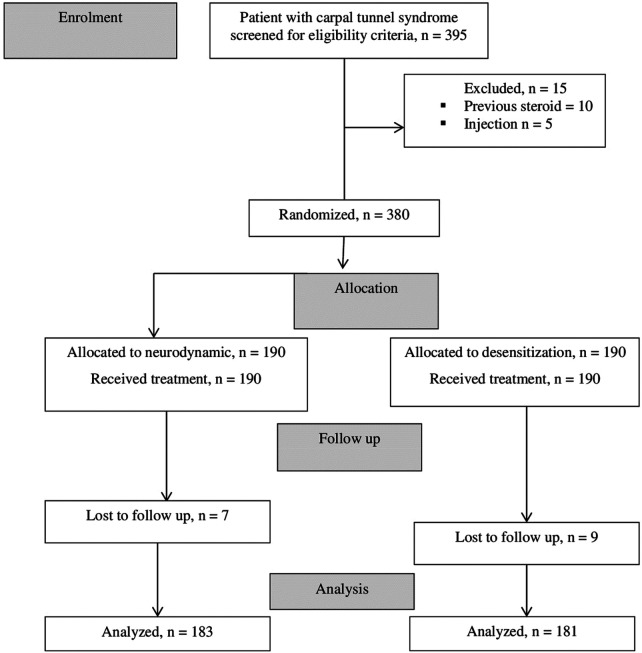
Flow diagram of phases through the clinical trial.

A total of 395 participants were included in this study. Of these, 15 did not meet the inclusion criteria (Figure 1). The 380 participants were randomly allocated to the neurodynamic technique or desensitization maneuver groups. After 3 months of treatment, 380 participants completed the analysis, with 190 participants in each group; after the 1-year follow-up, there were 183 participants in the neurodynamic technique group and 181 in the desensitization maneuvers group. Sixteen participants were lost to follow-up at 1 year. Participant characteristics are detailed in Table 1.

**Table 1:** Comparison between participant characteristics.

Characteristic	Neurodynamic group (*n*=190)	Desensitization group (*n*=190)	*P*-value [*P*-value]
Age (SD), years	53.3 ± 2.2	54 ± 1.3	*P* = 0.9123*
Body mass index (SD)	30 ± 3.2	29.3 ± 4.1	*P* = 0.7453*
Dominant hand			
Right *n* (%)	100 (53)	105 (56)	*P* = 0.43**
Left *n* (%)	90 (47)	85 (54)	
Symptomatic hand			
Right, *n* (%)	95 (50)	86 (46)	*P* = 0.37**
Left, *n* (%)	95 (50)	104 (54)	

* Chi-square test.

** Independent *t*-test.

At baseline, both groups were similar in terms of body mass index, symptomatic hand and hand dominance, and pain numerical rating scale (Table 2). Analysis revealed group differences after treatment, and the effect of therapy within the same group before and after treatment was significant (Table 2).

**Table 2:** Comparison before and after treatment in the nerve conduction study.

Examination	Group	Before treatment	After treatment	Effect of treatment
Sensory conduction (m/s)	Neuro dynamic	23.7 ± 8.2	36 ± 10.5	*P* < 0.001*
	Desensitization	24.1 ± 6.72	53.1 ± 11.0	*P* < 0.0001*
	Between groups difference	*P* = 0.9998	*P* < 0.001*	Significant
Motor conduction velocity (m/s)	Neuro dynamic	53 ± 2	52.4 ± 4	*P* < 0.01*
	Desensitization	50 ± 2	54 ± 2	*P* < 0.001*
	Between groups difference	*P* = 0.6223	*P* < 0.001*	Significant
Motor latency, *t* (ms)	Neuro dynamic	4.88 ± 1	4.42 ± 1.2	*P* < 0.01*
	Desensitization	5 ± 1.0	3 ± 0.33	*P* < 0.001*
	Between groups difference	*P* = 0.7811	*P* < 0.001*	Significant

* Statistically significant.

For the motor conduction velocity, the neurodynamic group was 52 ± 4 m/s before treatment and 53.1 ± 2 m/s after 3 months of treatment (*P* < 0.01). For the desensitization group, sensory conduction velocity was 50 ± 2 m/s before treatment and 54 ± 2 m/s after 3 months of treatment (*P* < 0.001). Analysis performed on motor conduction of the median nerve before treatment was not significantly different (*P* = 0.6223), although there was a difference after treatment (*P* < 0.001).

For motor latency, the neurodynamic group was 4.88 ± 1.0 m/s before treatment and 4.42 ± 1.2 m/s after 3 months of treatment (*P* < 0.01). In the desensitization group, motor latency was 5 ± 1 m/s before treatment and 3 ± 0.33 m/s after 3 months of treatment (*P* < 0.001). Analysis performed on motor latency of the median nerve before treatment was not significantly different (*P* = 0.7811), although there was a difference after treatment (*P* < 0.001).

The results of the assessment of the numerical pain rating scale by Analysis revealed group differences after treatment (*P* < 0.001), before and after treatment (*P* < 0.001), and at 1-year follow-up between the two groups (*P* < 0.001) (Table 3). Analysis on Boston Carpal Tunnel Questionnaire (symptoms severity scale) for the neurodynamic group was 3.4 ± 1.63 before treatment and 2.1 ± 0.32 after 3 months of treatment; after 1-year follow-up, this was 2.1 ± 0.03 (*P* < 0.001). For the desensitization group, this was 3.7 ± 1.65 before treatment and 1.02 ± 0.21 after 3 months of treatment; after 1-year follow-up, this was 1.0 ± 0.1 (*P* < 0.001). Analysis performed for the assessment of the Boston Carpal Tunnel Questionnaire (symptoms severity scale) before treatment showed no group difference (*P* = 0.8733), although there was a difference after treatment (*P* < 0.001) (Table 3).

**Table 3:** Comparison of numerical pain rating scale, Boston Carpal Tunnel Questionnaire, and cylindrical grip and pincer grip strength.

Examination	Group	Before treatment	After treatment	One-year follow-up	Effect of treatment
Numerical pain rating scale (0–10)	Neuro dynamic	6 ± 1	3.3 ± 2.3	3.7 ± 2	*P* < 0.001*
	Desensitization	6.66 ± 1	1 ± 67.0	1.1 ± 0.86	*P* < 0.0001*
	Between groups difference	*P* = 0.8753	*P* < 0.001*	*P* < 0.001*	Significant
Boston Carpal Tunnel Questionnaire symptoms severity scale	Neuro dynamic	3.2 ± 1.63	2.4 ± 0.3	2.3 ± 0.05	*P* < 0.001*
	Desensitization	3.9 ± 1.0	89.02 ± 0.11	99.0 ± 0.6	*P* < 0.001
	Between groups difference	*P* = 0.8733	*P* < 0.001*	*P* < 0.001*	Significant
Boston Carpal Tunnel Questionnaire, functional status scale	Neuro dynamic	3.5 ± 1.51	1.88 ± 0.14	2.1 ± 0.3	*P* < 0.001*
	Desensitization	3.4 ± 1.45	1.01 ± 0.11	1.0 ± 0.09	*P* < 0.001*
	Between groups difference	*P* = 0.9373	*P* < 0.001*	*P* < 0.001*	Significant
Cylindrical grip, kg	Neuro dynamic	19.4 ± 2.31	22.0 ± 2.5	NA	*P* < 0.432
	Desensitization	20 ± 1.0	26.0 ± 1.55		*P* < 0.001*
	Between groups difference	*P* = 0.9433	*P* < 0.001*	NA	Significant
Pincer grip, kg	Neuro dynamic	5.5 ± 0.4	6.88 ± 0.6	NA	*P* < 0.351
	Desensitization	5.9 ± 0.1	7.2 ± 0.55		*P* < 0.001*
	Between groups difference	*P* = 0.8933	*P* < 0.001*	NA	Significant

NA, not applicable.

* Statistical significance.

Analysis on the Boston Carpal Tunnel Questionnaire (functional status scale) for the neurodynamic group was 3.2 ± 1.51 before treatment and 2.3 ± 0.12 after 3 months of treatment; after 1 year of follow-up, this was 2.34 ± 0.3 (*P* < 0.001). For the desensitization group, this was 3.4 ± 1.45 before treatment and 1.01 ± 0.11 after 3 months of treatment; after 1-year follow-up, this was 1.0 ± 0.09 (*P* < 0.001). Analysis performed for the Boston Carpal Tunnel Questionnaire (functional status scale) before treatment was not significantly different (*P* = 0.9373), although there was a difference after treatment (*P* < 0.001) (Table 3). For the desensitization group, this was 21.5 ± 1.55 before treatment and 27 ± 2.1 after 3 months of treatment (*P* < 0.001). Analysis performed for the assessment of cylindrical grip before treatment was not significantly different (*P* = 0.9433), although there was a difference after treatment (*P* < 0.001) (Table 3).

Analysis on pincer grip for neuro dynamic group was not significant. For the desensitization group, this was 6.1 ± 0.75 before the treatment and 8.3 ± 0.31 after 3 months of treatment (*P* < 0.001). Analysis performed for the assessment of cylindrical grip before treatment was not significantly different (*P* = 0.8933), although there was a difference after treatment (*P* < 0.001) (Table 3).

Between-group effect sizes at post-treatment favored the desensitization group across all outcomes. The magnitude of effects was large for pain (Cohen's *d* = 1.36), very large for BCTQ symptom severity (*d* = 6.11) and functional status (*d* = 6.91), and very large for cylindrical grip strength (*d* = 1.92). The between-group effect for pincer grip was moderate (*d* = 0.56). These findings indicate not only statistical but also substantial clinical differences between interventions.

The reductions in pain exceeded established thresholds for minimal clinically important difference on the NPRS, and the improvements in BCTQ scores represent large, clinically meaningful gains in symptom severity and functional capacity.

## Discussion

This is the first prospective, randomized clinical trial with blinded outcome assessment to explore the long-term efficacy of neurodynamic techniques and desensitization maneuvers in the conservative treatment of patients with carpal tunnel syndrome. The results provide evidence for the efficacy of both types of treatment (neurodynamic techniques and desensitization maneuvers), but the desensitization maneuvers were superior in results, and statistically significant effects were obtained in nerve conduction study, pain and symptom severity, and functional status. There were also statistically significant effects on the assessment of muscle strength in cylindrical and pincer grip tests. Beneficial therapeutic effects were observed in both groups, but greater results were achieved in the desensitization maneuver group. Desensitization maneuvers were equally effective after treatment and 1-year follow-up in improving pain and function in women with carpal tunnel syndrome.

Many studies have assessed the use of neurodynamic techniques (5, 12, 13, 16), and others have assessed the use of desensitization maneuvers (8, 12, 17, 18), but none have compared both. Electrophysiological assessment in the form of a nerve conduction study is very sensitive in examination of the median nerve in cases of carpal tunnel syndrome. These tests can define the degree of demyelination and axonal loss that occurs during the diagnosis of carpal tunnel syndrome, and are useful in the assessment of nerve function and quantity of nerve damage because the findings could have implications for prognosis (2).

Sensory nerve conduction velocity, motor nerve conduction velocity, and motor latency were significant in both treatment groups before and after treatment. When both groups were compared after treatment, there were significant differences between the groups, and the desensitization maneuvers were superior in improvement, despite no significant difference between the groups before the treatment.

The use of neurodynamic techniques and desensitization maneuvers produced a significant reduction in pain; however, the effect was more pronounced in the desensitization maneuver group. The mechanism of decreased pain may be multifactorial due to decreased pressure in the carpal tunnel and decreased tissue edema. It is known that nerve compression can cause chronic inflammation that leads to carpal tunnel syndrome symptoms. Wolny and Linek (13) hypothesized that the use of neurodynamic techniques may increase blood supply, reduce mechanical irritation, and improve nerve sliding to improve physiological function. On the other hand, desensitization maneuvers were directed to the entire involved upper extremity according to the current nociceptive pain theories of carpal tunnel syndrome. Desensitization maneuvers of the central nervous system may be more effective than localized interventions targeting only the hands and/or wrists in this population, due to the pressure pain threshold decrease accompanied by pain intensity and symptoms this is supporting the role of peripheral drive to initiate and maintain central sensitization due to both central and peripheral sensitization mechanisms are involved at the same time in carpal tunnel syndrome (19).

The superior outcomes observed with desensitization maneuvers may reflect modulation of both peripheral nociceptive input and central sensitization processes. Manual therapy directed at cervical and proximal neural structures may influence descending inhibitory pathways and cortical representation of pain, thereby enhancing symptom resolution beyond localized neural mobilization alone.

The very large between-group effect sizes for BCTQ symptom severity and functional status suggest that desensitization maneuvers may exert broader therapeutic effects than localized neurodynamic mobilization, potentially through modulation of central sensitization in addition to peripheral neural and soft-tissue mechanisms.

The neurodynamic techniques group also experienced a decrease in symptom severity and an improvement in function, but the improvement in the desensitization maneuvers group was superior. This explains the great improvement in nerve conduction due to the impaired conduction velocity as a manifestation of carpal tunnel syndrome, and why this disappears following treatment (20).

No significant changes were observed in cylindrical and pincer grip after treatment in the neurodynamic group, in agreement with Wolny and Linek (13); there were significant changes in the desensitization group.

In the current study, the manual physical therapy approach included soft tissue mobilization and nerve/tendon gliding techniques directed at the entire involved upper extremity, according to current nociceptive pain theories on carpal tunnel syndrome. Traditionally, carpal tunnel syndrome has been considered a pathology associated with peripheral nerve lesions, but there is evidence suggesting that it is a more complex disorder with potential central sensitization processes (19).

It has been suggested that the desensitization maneuvers used in the current trial may have an impact on sensitization mechanisms. It is possible that manual physical therapy interventions have the potential to decrease sensitization, which could result in overall improved quality of life, contributing to a greater incremental quality of life and cost-effectiveness (17).

In the long term, manual therapy, including desensitization maneuvers of the central nervous system, results in similar outcomes and surgery rates compared with surgery in women with carpal tunnel syndrome (18).

The study observed that the desensitization maneuvers and neurodynamic techniques were effective, except the neurodynamic techniques did not achieve any changes in the cylindrical grip and pincer grip, which agrees with the study by Wolny and Linek (13), who stated that the use of neurodynamic techniques in conservative treatment for mild and moderate forms of carpal tunnel syndrome has significant therapeutic benefits, but no change in a cylindrical and pincer grip (13). This study provides promising results for desensitization maneuvers of the central nervous system in the treatment of carpal tunnel syndrome at a 1-year follow-up.

There were limitations of this study. There was no control group; all participants were female and had moderate severity of carpal tunnel syndrome. Also, the lack of a nerve conduction study at the 1-year follow-up and the lack of measurement of the cervical range of motion limited the results. The role of psychological variables such as mood disorders or sleep disturbances was not considered. Additionally, the provocative tests used in the physical examination, such as the Phalen or Tinel test data, at any follow-up period, were not reassessed, which could also elucidate potential between-group differences. It is unknown if a greater number of sessions would reveal differences between the interventions. Further research is needed to replicate the current findings in a different sample of individuals with carpal tunnel syndrome. Additional research may attempt to determine the optimal manual therapy dosage, such as the frequency of the intervention. An important limitation is the difference in treatment frequency between groups. Although based on established protocols, unequal session numbers may have contributed to outcome differences. Future trials should compare these interventions using dose-matched designs to isolate technique-specific effects.

## Conclusion

Both neurodynamic techniques and desensitization maneuvers produced significant improvements in women with moderate carpal tunnel syndrome. Desensitization maneuvers demonstrated superior short- and long-term outcomes in pain, functional status, and nerve conduction. These findings support the incorporation of centrally oriented manual therapy strategies in the conservative management of CTS; however, further dose-matched randomized trials are warranted.

## Data Availability

The raw data supporting the conclusions of this article will be made available by the authors, without undue reservation.
